# Characterization and genome sequencing of two *Propionibacterium acnes *phages displaying pseudolysogeny

**DOI:** 10.1186/1471-2164-12-198

**Published:** 2011-04-19

**Authors:** Rolf Lood, Mattias Collin

**Affiliations:** 1 Department of Clinical Sciences, Division of Infection Medicine, BMC-B14, Lund University, SE-221 84 Lund, Sweden

## Abstract

**Background:**

*Propionibacterium acnes *is a Gram positive rod inhabiting the human skin that also infects orthopaedic implants and is associated with acne vulgaris. Previously, one lytic bacteriophage, PA6, from *P. acnes *has been sequenced and partially characterized. We recently isolated several inducible phages from *P. acnes *classified as Siphoviruses based on morphology and partial genome sequencing.

**Results:**

In this study we sequenced the inducible *P. acnes *phages PAD20 and PAS50, isolated from deep infection and from skin, respectively. The genomes of PAD20 and PAS50 are 29,074 and 29,017 bp, respectively, compared with the 29,739 bp of PA6. The phage genomes have 87.3-88.7% nucleotide sequence identity. The genes are divided into clusters with different levels of similarity between the phages. PAD20 and PAS50 share four genes encoding identical amino acid sequences. Some deletions and insertions in the genomes have occurred, resulting in lack of genes, frame shifts, and possible regulatory differences. No obvious virulence factor gene candidates were found. The phages are inducible, but bacteria can be cured of phages by serial colony isolations and lose their phages during stationary phase, but are still sensitive to new phage infections. Construction of a phylogenetic tree based on more than 459 phage genomes, suggested that *P. acnes *phages represent a new lineage of Siphoviruses.

**Conclusions:**

The investigated *P. acnes *Siphovirus genomes share a high degree of homology to other *P. acnes *phages sequenced, but not to genomes of other phages isolated from *Propionibacteria*. The phage genomes are not integrated in the bacterial genome, but instead, most likely have a pseudolysogenic life cycle.

## Background

*Propionibacterium acnes *is a Gram positive rod normally found on human skin [1], but has also been isolated from infections of orthopaedic implants [2,3], and has been suggested to be involved in the development of acne vulgaris. Recently, the first genome of a *P. acnes *strain was sequenced [4] and several putative virulence factors were identified [5]. The sequenced *P. acnes *genome only contains one cryptic prophage, but it has earlier been shown that some *P. acnes *strains are carriers of inducible phages [6]. Other groups have also studied *Propionibacterium *phages both in order to establish a tool to differentiate between serotypes, but also to further characterize phages mainly infecting bacteria important for the dairy industry [7-16].

Recently, Farrar *et al*, sequenced the first genome of a *P. acnes *phage, PA6 (GenBank accession no. DQ431235) [17]. The genome consists of 29,739 bp dsDNA with 48 putative coding genes. The phage was determined to be a lytic phage since no integrase or repressor could be detected in the genome. Eighteen of the genes were assigned predicted functions based on their similarities to other known phage genes. Recently, we isolated and characterized inducible phages from *P. acnes *isolated from both deep infections and from skin of healthy individuals [6]. By comparing the genomes of these different phages, a better understanding of how the phage influences its host and an insight in the great genetic diversity seen among phages, as shown by other comparative studies on phage genomes can be achieved [18,19].

Phages can enter two principally different life cycles, e.g. a lytic life cycle or a lysogenic cycle. In the lysogenic cycle most phages integrate their genome into the host genome. This is most often regulated by a phage-borne integrase, but there is also a recent report of a filamentous phage, CTXϕ from *Vibrio cholerae*, that uses the host recombinases XerC and XerD to integrate into the host genome [20,21]. Furthermore, there are a few phages known to form stable linear or circular plasmids as a part of their integration mechanism [22-24], but most known temperate phages encode their own integrases.

Other than those two well-defined phage life cycles, phages are also known to be able to interact with their host in other life cycles, termed as persistent infections, pseudolysogeny, carrier-state, and chronic infections [25,26]. Those phage life cycles, even though common, are less defined compared to the lytic and lysogenic life cycles. Common for all states, except the chronic infection, is that the bacteria carry the phage DNA, but the DNA is not integrated into the bacterial chromosome, but rather exists as a plasmid. The chronic infection is characterized by the release of phages from the bacteria without causing lysis [25].

In this study, we have sequenced the genome of one phage induced from a *P*. *acnes *strain isolated from deep infection (PAD20), and the genome of one phage induced from a *P. acnes *strain isolated from skin (PAS50), and we have compared the two genomes with the reported lytic phage PA6 [17]. The phages have earlier been shown to have dissimilarities in the genes encoding a putative major head protein and an amidase [6], but no other genes have been examined. This offers an opportunity to investigate if phages isolated from *P. acnes *contribute to the virulence seen in different strains of *P. acnes*, and to examine the genetic diversity seen among phages.

## Results and discussion

### Genomic organization and homology

**Figure 1:**
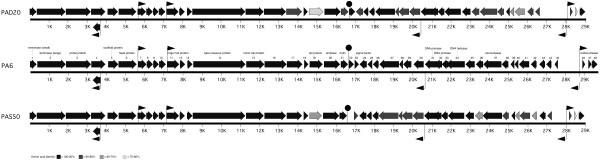
**Genomic organization of the *P. acnes *phages**. Schematic representation of the genome organization of *P. acnes *phages PAD20, PA6 and PAS50 and some functional assignments. The arrows indicate the different ORFs and the direction of transcription. The colours of the ORFs indicate the homology of phage PAD20 and PAS50 to PA6. Putative sigma70-promoters and transcriptional terminators are indicated.

The genomic organization of the three examined phages are very similar, even though some genes differ (see Figure 1). Both phages have a 13 bp ssDNA extension at both ends of the genome, identical to that of phage PA6. These extensions enable the formation of potential concatamers and circularization of the phage genome. The genome of PAD20 is 29,074 bp and has a GC content of 54.1%, compared to the genome of PAS50 that is 29,017 bp and has a GC content of 53.9%. PAD20 has two larger deletions in the genome compared to PA6. The first is a 226 bp deletion resulting in the deletion of the main part of gp22 and thereby in a frame shift giving rise to a combination of the gene products Gp22 and Gp23 (Gp22.23, see additional file 1). The second larger deletion is a 378 bp deletion of the entire gp42 gene. PAS50 has two larger deletions and one insertion. The first deletion in PAS50 is a 207 bp deletion resulting in the deletion of the gp45 gene, and a second deletion of almost 500 bp in a non-coding region, corresponding to bp 27,932-28,419 in PA6. The insert in PAS50 is an 84 bp insertion in gp37 resulting in a 28 amino acid larger exonuclease.

To further investigate the genomic organization of the different *P. acnes *phages, the genomes were screened for putative sigma70-dependent promoters and transcriptional terminators (see Figure 1 and Table 1). Several putative promoters were detected in all phages upstream of gp7, gp11 and gp46, as well as upstream of gp45 and gp30, potentially enabling the phage to transcribe different parts of the genome at different stages of infection. One of the promoters with the highest score was upstream of a hitherto unannotated ORF (3693-3301, gp50). However, the predicted gene product does not show any similarity to known sequences or domains.

**Table 1:** Putative regulatory elements

Phage	Position	gp	Sequence	Score
Promoters				

PA6	5620-5655	gp7	**GTGCAG**GGGGTGGTGTTGAT**GGGTATCAT**TTTGAAG	73/16

PA6	7124-7159	gp11	**TTGATA**TTGTTGTTTGTTTT**TTGTTTGAT**ATTGTTT	33/58

PA6	28708-28742	gp46	**TTAAAG**CTTATAGGTATAA**TAATATAAT**ATAAGTA	68/31

PA6	3740-3704	gp50	**TAGTAA**GTGCCCTCTCTTTTG**TCGTATAAT**CCGACCT	80/10

PA6	20693-20656	gp30	**TTGACA**CAAGCACTAGACACAG**TGCTACAAT**AAAACCA	72/66

PA6	28763-28724	gp45	**TTTATA**ACTTATAAGCTTTAATAC**TTATATTAT**ATTATTA	61/39

PAD20	5618-5653	gp7	**GTGCAG**GGGGTGGTGTTGAT**GGGTATCAT**TTTGAAG	73/16

PAD20	7104-7140	gp11	**CTGAGT**AAACGTATTTGTTTT**TGTTTTAAT**GTAATTG	55/3

PAD20	28024-28063	gp46	**TTAAAG**TTAACCATCAGTCTTAAA**CTTTAATAT**TATAACC	61/31

PAD20	3738-3704	gp50	**GTGAGT**GCCCTTTCTTTTG**TCGTATAAT**CCGACCT	80/5

PAD20	20473-20436	gp30	**TTGACA**CAAGCCCTCAACACAG**TGCTACAAT**AAAACCA	72/66

PAD20	28084-28048	gp45	**TTATAA**GCTTTAATACTTATA**GGTTATAAT**ATTAAAG	90/24

PAS50	5615-5650	gp7	**GTGCAG**GGGGTGGTGTTGAT**GGGTATCAT**TTTGAAG	73/16

PAS50	7101-7137	gp11	**CTGAGT**AAACGTATTTGTTTT**TGTTTTAAT**GTAATTG	55/3

PAS50	27980-28014	gp46	**TTAAAG**CTTATAGGTATAA**TAATATAAT**ATAAGTA	68/31

PAS50	3740-3704	gp50	**TAGTAA**GTGCCCTTTCCTTAG**TCGTATAAT**CCGACCT	80/10

PAS50	20641-20604	gp30	**TTGACA**CAAGCCCTCAACACAG**TGCTACAAT**AAAACCA	72/66

PAS50	28035-27996	gp45	**TTTATA**ACTTATAAGCTTTAATAC**TTATATTAT**ATTATTA	61/39

Terminators				MFE

PA6	16665-16713	gp21	**GTGCCCCAGCGG**TGCTG**CCAC**GATTGT**GTGG**TGGTTG**CCGCTGGGGCAC**	-39.9

PAD20	16682-16730	gp21	**GTGCCCCAGCGG**TGCTG**CCAC**GATCGT**GTGG**TGGTTG**CCGCTGGGGCAC**	-39.9

PAS50	16604-16652	gp21	**GTGCCCCAGCGG**TGCTG**CCAC**GATCGT**GTGG**TGGTTG**CCGCTGGGGCAC**	-39.9

The gp number represents the ORF directly after the regulatory element. Bold letters indicate the putative -10 and -35 site of the promoters, and the palindromes in the terminators. The score is given as -10/-35 for the promoters based on BPROM as described in Methods, and as minimal free energy (MFE) in kcal/mol for the terminators.

Except for the six identified promoters in each genome, one putative transcriptional terminator was also identified (see Figure 1). The putative terminators are placed immediately downstream of the amidase and the holin in the lysis gene cassette.

Even though most of the genes are very similar in the examined phages, a certain pattern of similarity between gene clusters can be observed. Packaging genes and putative head genes have a higher similarity between PAD20 and PAS50 than between the other examined *P. acnes *phage groups. Other than the clusters mentioned above, gp27-gp48 also shows the highest similarity between PAD20 and PAS50, except for gp42 and gp43. This exception is mainly due to the deletion of gp42 in PAD20. Furthermore, most tail genes and genes involved in the lysis of the host have the highest similarity between PA6 and PAS50. The overall genome nucleotide similarity is very high between all phages examined (PA6/PAS50 - 88.7%, PAD20/PAS50 - 87.8%, and PA6/PAD20 - 87.3%).

By searching for homologues corresponding to PAD20 and PAS50 proteins using BLASTp [27], most hits were in the *Actinobacteria *phylum, with *Streptomyces*, *Mycobacterium *and phages infecting *Mycobacterium*, *Propionibacterium*, *Nocardia*, and *Bifidobacterium *(see Table 2). This is not surprising since these species are closely related and thereby also might have similarities among their phages. Most hits were on bacterial proteins, most likely indicating that these bacterial strains have prophages that have not yet been examined. More interestingly, several phage proteins showed similarity to phages isolated from low G+C bacteria as *Streptococcus *and *Lactococcus *(see Table 2). These species are not very closely related to *P. acnes*, but the results might indicate that their phages have had several possibilities to exchange genes. Surprisingly, there were no hits on any other *Propionibacterium *phages or prophages. If this merely reflects the lack of sequenced phages from *Propionibacteria *and bacterial genomes in the *Propionibacterium *genus, or indicate that phages isolated from *P. acnes *are distinct from those infecting other *Propionibacteria *is unknown. Furthermore, the putative cryptic prophage in *P. acnes *strain KPA171202 [5] does not show any similarity to PAD20, PAS50 or PA6. However, most recently, Machuca *et al *[28] isolated a phage infecting *Fusobacterium nucleatum*. This phage was partially sequenced (390 bp), and showed 90% sequence identity to parts of gp3 (portal protein) and gp4 in PA6. However, the whole phage genome of the *Fusobacterium *phage needs to be sequenced to conclude how related this phage is to *Propionibacterium *phages.

**Table 2:** Phage protein similarities

Gene	Location (PAD20/PAS50)	Function	Hits	E-value PAD20	E-value PAS50
gp1	2-373 2-373	Small terminase	*Mycobacterium *phage Brujita gp1	8e-06	3e-05

			*Streptomyces *phage VWB gp32	3e-05	5e-05

gp2	378-1889 378-1889	Large terminase	*Bifidobacterium *hypothetical protein BIFDEN_01707	9e-79	2e-78

			*Streptococcus *phage terminase	2e-37	2e-37

			*Lactococcus *phage r1t putative terminase	5e-37	4e-36

gp3	1886-3211 1886-3211	Portal protein	*Corynebacterium *hypothetical protein cur_1413	2e-70	6e-69

			*Mycobacterium *phage Lockley gp12	3e-36	5e-33

			*Lactococcus *phage phiLC3 putative portal protein	3e-29	2e-28

			*Streptococcus *phage SM1 gp34	7e-27	3e-26

gp4	3218-3973 3218-3973		*Mycobacterium *phage TM4 gp6	2e-06	3e-05

			*Rhabdovirus *nucleocapsid protein	7.3e-02	2e-01

gp5	4084-4638 4081-4635	Scaffold protein	*Strongylocentrotus *hypothetical protein	5.9e-02	1.1e-01

			*Mycobacterium *phage Tweety gp5	9.5e-01	9e-03

			*Streptococcus *phage SM1 gp39	1.1e-00	1.1e-00

gp6	4645-5592 4642-5589	Major head protein	*Mycobacterium *phage Che9d gp7	8e-49	7e-50

			*Streptococcus *phage SM1 gp40	1e-30	2e-30

			*Lactococcus *phage phiLC3 MHP	2e-30	1e-29

gp7	5636-6097 5633-6094		*Mycobacterium *phage Halo gp9	5e-26	4e-26

gp8	6099-6446 6096-6443				

gp9	6453-6743 6450-6740		*Streptococcus *conserved hypothetical protein	8.3e-01	2.5e-00

gp10	6740-7111 6737-7108		Amidase domain	4.7e-01	6.1e-01

			*Vibrio *MoxR-related protein	2.1e-00	9.7e-01

gp11	7164-7793 7161-7799	Major tail protein	*Leifsonia *structural phage protein	4e-21	6e-21

			*Streptococcus *major tail protein	2e-10	3e-09

			*Lactococcus *phage phiLC3 MTP	5e-04	1e-04

			*Streptomyces *phage major tail protein	2e-03	2e-03

			*Mycobacterium *phage Wildcat gp35	1.2e-02	7e-03

gp12	7821-8114 7827-8123		*Ralstonia *DNA topoisomerase IV subunit A	3.7e-01	-

			*Methylocella *peptidase M23	1.5e-00	3e-00

gp13	8213-8500 8222-8509		Putative transmembrane protein pfam 10271	1.8e-01	-

gp14	8508-11273 8517-11282	Tape measure protein	*Atopobium rimae *gp14	5e-51	3e-54

			*Bifidobacterium *phage related protein	7e-32	1e-40

			*Microbacterium *phage Min1 probable	2e-28	2e-28

			tape measure protein		

			*Mycobacterium *phage Che9d gp17	3e-28	4e-28

gp15	11289-12230 11298-12239	Minor tail protein	*Streptomyces *hypothetical protein SAV_5507	9e-06	2e-08

			*Leifsonia *phage.related protein Lxx24170	1e-03	1e-03

gp16	12238-13395 12247-13404		*Bacillus *phage minor structural protein	4e-05	4e-04

			*Streptomyces *hypothetical protein SAV_5506	5e-04	4e-05

			*Salinospora *hypothetical protein Strop_0531	2e-03	1e-05

			*Geobacillus *phage-related protein GK0542	4e-03	1e-03

gp17	13413-14231 13421-14239		H-type lectin domain	3.9e-02	1e-03

gp18	14300-14539 14308-14553				

gp19	14599-15339 14607-15269	Tail protein	*Streptococcus *collagen-like surface protein	2e-13	1e-14

			Collagen domain	4e-03	2.3e-02

gp20	15389-16252 15318-16175	Amidase	*Propionibacterium *putative amidase	1e-48	3e-50

			*Mycobacterium *phage Chah gp50	2e-07	6e-06

			Amidase 2 domain	1e-07	1e-07

gp21	16265-16669 16188-16589	Holin	*Mycobacterium *phage Tweety gp32	1.5e-00	6.6e-00

			*Lactococcus *phage r1t holin	1.9e-00	2.2e-00

gp22.23	16676-17005 -				

gp23	- 16941-17174				

gp24	17428-17033 17591-17202	Sigma factor	Sigma 70, region 4	9e-03	1.9e-02

			HTH transcription regulator	2.2e-01	1.8e-01

gp25	17706-17425 17879-17595		*Prochlorococcus *phage P-SSM4 gp7	7.1e-00	9.8e-01

gp26	18041-17721 18212-17892		*Prochlorococcus *hypothetical protein A9601_14091	6.7e-00	3.3e-00

gp27	19098-18052 19266-18220		*Mycobacterium *phage TM4 gp36	1e-27	6e-27

			*Nocardia *hypothetical protein nfa39310	1e-23	3e-22

gp28	19289-19095 19457-19263				

gp29.1	19836-19273 20004-19441				

gp30	20396-19833 20564-20001		Hyaluronidase domain	3.9e-00	2.1e-01

			*Aspergillus *hypothetical protein An14g04790	1.1e-01	9e-01

gp31	21112-20441 21277-20609	DNA primase	*Salinospora *hypothetical protein Strop_0519	3e-31	4e-27

			*Mycobacterium *phage Solon gp53	4e-22	1e-20

			Topoisomerase primase	2e-05	1e-04

gp32	21350-20949 21515-21114	DNA primase	DnaG, DNA primase	1e-08	3e-05

			Marine gamma proteobacterium DNA primase	4e-05	3e-03

			*Prochlorococcus *DNA primase	9e-05	8e-03

gp33	21666-21310 21831-21475		NERD (nuclease related domain)	1.1e-02	1.3e-02

			LlajI restriction endonuclease	1.6e-01	1.6e-01

			Holliday junction resolvase	1.7e-01	4.1e-01

			*Mycobacterium *phage Cjw1 gp87	1.6e-00	4.3e-01

gp34	22529-21666 22694-21831	DNA helicase	*Salinospora *hypothetical protein Strop_0521	2e-47	3e-45

			*Mycobacterium *phage KBG gp64	2e-15	5e-15

			Multidrug Resistance Protein-like transporter	7e-06	3e-04

			DnaB helicase C terminal domain	2e-04	2e-03

gp35	23036-22572 23201-22737		*Verrucomicrobium *PEP synthase	3.3e-01	1.1e-00

			*Prochlorococcus *possible ABC	1.8e-00	1.8e-00

			transporter related		

gp36	23488-23078 23652-23242				

gp37	24432-23503 24680-23649	Exonuclease	*Mycobacterium *phage Bethlehem gp69	2e-16	6e-16

			*Mycobacterium *phage U2 putative RecB family exonuclease	2e-13	6e-13

gp38	24785-24432 25033-24680		*Bartonella *phage-related protein Btr_0991	6e-11	3e-13

			*Mycobacterium *phage TM4 gp52	8e-10	4e-11

gp39	25094-24891 25342-25139				

gp40	25318-25091 25566-25339				

gp41	25876-25343 26124-25591		*Alkalilimnicola *outer membrane lipoprotein LolB	2.6e-02	-

gp42	- 26492-26208		*Rhodobacterales *methylamine utilization protein MauG	-	2.3e-01

gp43	26311-26000 26932-26621				

gp44	26653-26387 27274-27008				

gp46	28207-28362 28150-28305				

gp47	28492-28626 28435-28569		*Bordetella *probable transcriptional regulator	4.8e-00	4.8e-00

gp48	28723-29025 28663-28968	Endonuclease	HNH endonuclease	5e-07	1e-06

			*Mycobacterium *phage TM4 gp92	1e-03	3e-03

gp49	28626-75		Topoisomerase II-associated protein PAT1	3.9e-02	5e-03

			*Zaire Ebola Virus *VP30	-	1.7e-01

			*Zaire Ebola Virus *minor nucleoprotein	-	1.9e-01

gp50	3693-3301 3693-3301				

Phage PAD20 and PAS50 protein sequences were screened for similar proteins using BLAST. All proteins showed the highest similarity to *P. acnes *phage PA6. Those hits were not further analyzed since they only indicate that the phages are most related to each other.

### Genes encoding packaging proteins and structural proteins

The packaging and structural proteins (Gp1-Gp19) are generally very well conserved, with high sequence similarities between all three phages, ranging from 89.7% to 99.3% amino acid identity (see Figure 1 and additional file 2) with one exception - gp19. Gp19 has 67.1% to 77.4% amino acid identity and is predicted to function as a tail protein, with high similarity to collagen. This protein is likely to be involved in host specificity, so a more diverged gene would imply that the phages might have differences in host specificity. The difference in host specificity between PAD20 and PAS50 is however not very pronounced, since both PAD20 and PAS50 are equally potent to initiate lysis in most examined *P. acnes *isolates, even though some isolates can be used to differentiate the phages [6]. This does not rule out that a larger screen would detect more differences in host specificity and more clearly point out the genetic varieties that determine the host specificity.

Another gene of particular interest is gp17 encoding a protein that has not yet been assigned a putative function. Gp17 has a H-type lectin domain with E-values ranging from 0.001-0.04 in the phages examined. Even though the E-values are rather low, they might still give an indication of the putative function. H-type lectins are known to interact with carbohydrates, and the genomic position of gp17, close to genes encoding tail proteins, makes it possible that this protein is also a tail protein interacting with carbohydrates in order to bind to certain structures and potentially mediate a first binding site for the phage.

### The lysis mechanism

All three phages encode an amidase, Gp20, with very high similarity to a *P. acnes *autolysin. They also encode a putative holin (Gp21) with more pronounced sequence divergence. Phage PA6 and PAS50 have 94% amino acid identity in the holin protein, while PA6 and PAD20 only have 84.3% amino acid identity. Interestingly, phage PAD20 has a deletion in gp22 resulting in a mainly deleted gp22 and a new open reading frame encoding a protein with similarities to both Gp22 and Gp23 in PA6 (see additional file 1). gp22 will not be fully translated in PAS50 due to two internal stop codons.

### Identical proteins and putative virulence factors

Several genes (gp39, gp40, gp44 and gp47) encode identical amino acid sequences in PAD20 and PAS50, but none of these proteins have predicted functions, nor any significant BLASTp hits, so these genes seem to be specific for *P. acnes *phages, and might, due to their sequence similarity be a good tool to use in phylogenetic analyses of phages isolated from *P. acnes*. gp29, a gene in PA6, is absent in both PAD20 and PAS50, but another ORF in the same region (gp29.1, 19,490-20,056 in PA6) exists in all three phages and might thereby be more likely to be a true coding gene. However, none of the putative gp29.1 genes have similarities to other genes, neither has gp29 in PA6.

Some proteins did get BLAST hits indicating that the proteins could be putative virulence factors. Among those are Gp34 that is annotated as a DNA helicase due to a DnaB helicase domain (E-value 2e-04 for PAD20). More interesting, this protein also has a Multidrug Resistance Protein-like transporter domain (E-value 7e-06 for PAD20). This domain is less pronounced in PAS50 (E-value 0.15) and PA6 (E-value 0.001). PAS50 also has a weak hit (E-value 0.21) in Gp30 on a hyaluronidase. However, due to the low similarities to other proteins, and thereby the low E-values, we can not assign a function to the phage proteins based on the similarities to other domains, only determine which domains they are most similar to.

The phages might also be able to circularize and form concatamers due to the ssDNA extensions on each side of the genome. When circularized, a new putative gene (gp49) can be transcribed. This gene encodes a protein with some similarity to different nucleoproteins from Ebola virus, and has a topoisomerase II-associated protein PAT-1 domain (E-value 0.005 for PAS50). Thus, this gene can only be transcribed when the phage has circularized and potentially integrated, or formed concatamers.

### Identification of phage proteins

**Figure 2:**
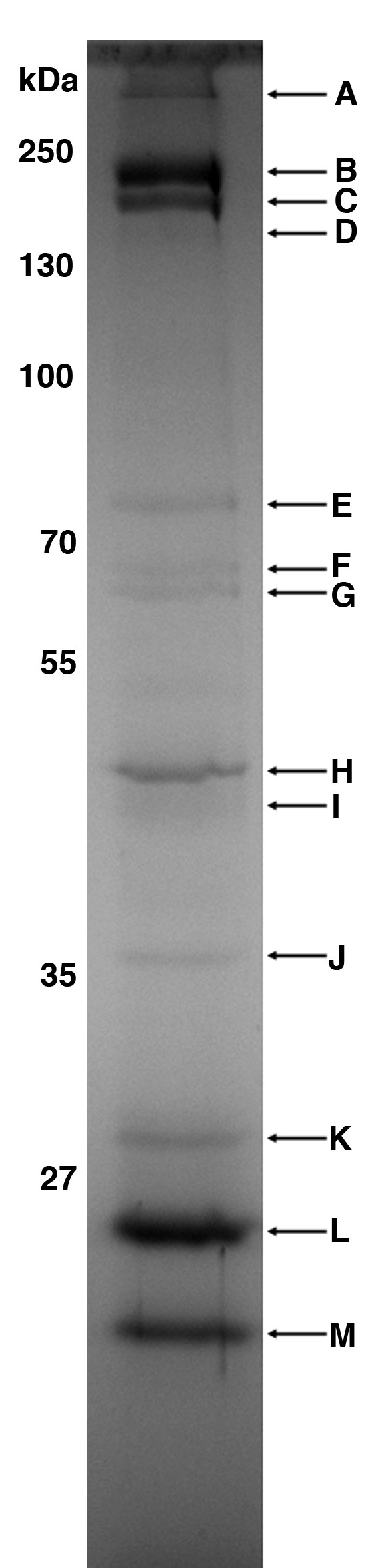
**SDS-PAGE on phage proteins**. Phages were purified using PEG precipitation and gel filtration. The purified phage proteins were separated on a 10% SDS-PAGE. The protein pattern of phage PAD20 was identical to that of PAS50. Marked bands (arrow) were further analysed with MS/MS. Almost all bands were identified as phage proteins (see Table 3).

To verify that some of the genes encoded proteins, PAD20 was precipitated with PEG 6000, the proteins were separated on a polyacrylamide gel, and then analyzed with MS/MS. All visible bands but band F were identified (see Figure 2 and Table 3). One of the bands, band E, was identified as a *P. acnes *succinate dehydrogenase flavoprotein subunit, thus indicating that this protein exists as a large complex, thereby being eluted in the same fractions as the whole phage particles during the FPLC gel filtration. Other than the *P. acnes *flavoprotein, all proteins were identified as phage proteins. Bands A-D were all identified as the major head protein of PAD20 (Gp6), which has a calculated molecular weight of approximately 33 kDa. However, the bands A-D have a much higher molecular weight, starting from approximately 160 kDa for band D, reaching over 500 kDa for band A. Interestingly, we did not get any other hits on the major head protein with lower molecular mass proteins, indicating that these proteins tend to form complexes of 6-8 monomers, based on the apparent molecular weight, that are not disrupted even when boiling the samples with SDS and reducing agents. A similar phenomenon has been described before with the *Enterobacteria *phage HK97, which has complexes of covalent linked major head proteins, and not a single major head protein exist as a monomer [29]. Both the N-and C-terminal part of the major head protein were covered, showing that this protein is not processed after its translation, as some major head proteins require in order to establish their final conformation.

**Table 3:** Phage and bacterial proteins identified by MS/MS

Band	Protein	Estimated molecular mass (kDa)	Molecular mass (Da)	Queries matched	Score	Sequence coverage (%)
A	Gp6 phage PAD20	>500	32911	11	357	36

B	Gp6 phage PAD20	230	32911	8	294	36

C	Gp6 phage PAD20	200	32911	10	284	36

D	Gp6 phage PAD20	160	32911	3	50	11

E	Succinate dehydrogenase flavoprotein subunit	75	76314	5	159	9

F	N.D.	68				

G	Gp14 phage PA6	65	94408	2	49	3

H	Gp3 phage PA6	48	48363	7	221	18

I	Gp16 phage PA6	44	43045	2	101	8

J	Gp15 phage PA6	36	34900	5	105	25

K	Gp17 phage PA6	29	28835	1	15	3

L	Gp11 phage PA6	26	22831	3	77	16

M	Gp11 phage PA6	23	22831	4	113	15

Other proteins that were identified were Gp14 (tape measure protein), Gp3 (portal protein), Gp16, Gp15 (minor tail protein) and Gp11 (major tail protein. Furthermore, band K did not result in a significant hit. However, the peptide with the highest score was similar to Gp17 from *P. acnes *phage PA6. This is further strengthen by that the apparent molecular weight of the protein band (29 kDa) is close to the theoretical molecular weight (28,835 Da). Thus, all phage proteins identified seem to be structural proteins, dominated by Gp6 (major head protein) and Gp11 (major tail protein).

### Phage life cycle

The phages used in this study were originally isolated by mitomycin C treatment of *P*. *acnes *isolates. However, those phages were able to infect their host strains, and thus did not confer superinfection immunity [6]. This data made us conclude that the phages were lysogenic. A similar phenomenon has been reported for *Leuconostoc oenos *phages, where more than 65% of the bacterial isolates could be induced with mitomycin C and where the phages were able to infect their host strains [30].

To further investigate how stable the interaction between the *P. acnes *phages and the bacterium was, we performed ten serial single-colony isolations and then screened for phages using a PCR assay. All examined colonies had been cured from phages (data not shown), indicating that the phages are not true lysogens. This ability to cure the bacterial isolates from phages suggests that the interaction between the phage and bacterium rather might be pseudolysogenic [31-33]. The inability to cure bacteria from phages was one of the criteria used to determine that phages from the *P*. *acnes *related bacterium *Nocardia erythropolis *were lysogenic, due to that the spontaneous curing of phages was less than 0.4% after 5 serial single-colony isolations [34]. These phages were also induced with mitomycin C, but they all showed superinfection immunity. It has also been shown in several experiments that the environment may influence the virus-host interaction. This is evident in the case of phages infecting the archeal species *Halobacterium*, where the NaCl concentration determines if the phages enter a lytic cycle, or cause a persisting infection. At low NaCl concentration, the phages effectively kill the host, while at higher concentrations of NaCl the lysis is delayed for several bacterial generations [35-37], leading to a persistent infection.

**Figure 3:**
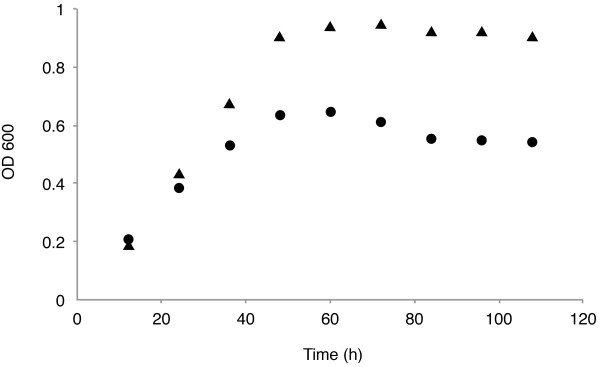
**Growth curve of the *P. acnes *isolates used**. *P. acnes *isolates AD20 (triangles) and AS50 (circles) were cultured in BHI, and OD_600 _was measured every 12 hours. The figure is a representative growth curve out of three independent experiments.

To further investigate if *P. acnes *might harbour pseudolysogenic phages, single colonies from the *P. acnes *isolates AD20 and AS50 were inoculated into BHI broth and different parameters such as OD_600 _and pfu were measured two times a day (see Figure 3 and Table 4). No free infectious phages were detected at any time point, indicating that if any phages were spontaneously induced, their number have not exceeded the detection limit of 100 pfu/ml. However, at time points corresponding to mid-log phase and late-log phase, both AD20 and AS50 had 10-60% colonies harbouring phage DNA, as judged by PCR amplification of the major head gene (see Table 4). However, this carriage of phages is lost during the stationary growth phase, thus indicating that the phage DNA is not being replicated at this time point. Furthermore, the cells at the various time points were sensitive to PAD20 and PAS50 infection, indicating that the loss of phages were not due to the development of resistance (data not shown). This implies that the phages are not strictly lytic, neither strictly lysogenic.

**Table 4:** Carriage of phages during growth of *P.acnes*

Time (h)	AD20	AS50
12	0/10	0/10

24	1/10	0/10

36	1/10	6/10

48	4/10	0/10

60	0/10	0/10

72	0/10	0/10

84	0/10	0/10

96	0/10	0/10

108	0/10	0/10

Much of the data indicates that the *P. acnes *phages might have a pseudolysogenic cycle, which is also supported by the genetic information. There are no genes on the phage genomes with similarities to known integrases or repressors. It is, however, known that certain filamentous phages can integrate their genomes using host recombinases [20,21]. However, when extracting *P. acnes *chromosomal DNA, phage DNA is lost, as judged by the absence of a positive PCR reaction for the phage major head gene (data not shown), indicating that the phage indeed does not integrate. Thus, it seems like *P. acnes *phages can persist as extra-chromosomal plasmids, however when in this state, they are not replicating, since they easily are cured after 10 serial single-colony isolations.

### Phage phylogenetic analysis

**Figure 4:**
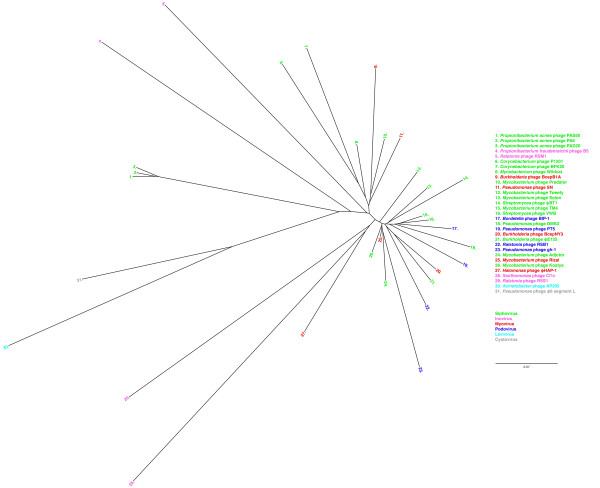
**Phages isolated from *P. acnes *represent a distinct phylogenetic lineage of Siphoviruses**. More than 450 phage genomes were aligned using a progressive Mauve alignment. For visualization, 28 of the phages representing different clusters of most closely related phages to *P. acnes *phages were realigned using the same settings. The different colours represent different families of phages, as indicated by the key.

Bacteriophages can be divided into different groups based on different criteria as host range, morphology and genetic material. Earlier attempts to classify phages according to their proteome have been conducted by Rohwer *et al *using 105 phage genomes [38]. We aligned 453 bacteriophage genomes, including PAD20 and PAS50, using the progressive Mauve algorithm [39] in order to investigate how the *P. acnes *phages were related to other phages. Mauve recognizes conserved nucleotide segments in the genomes, but has the advantage that it can rearrange the order of these segments, thereby also identifying genome rearrangements [40]. However, Mauve has several limitations when it comes to comparing several diverged phage genomes, due to that Mauve works best with related organisms, and can not identify conserved regions shared only by a fraction of the genomes. Thus, aligning the genomes using Mauve will not generate a reliable phylogenetic tree. Instead, we used the progressive Mauve algorithm, which is developed to be able to align more diverged genomes with less than 50% nucleotide identity. Our results are in good agreement with the results obtained by Rohwer *et al*, but some of the phages are placed differentially. The Sk1-like Siphoviruses with *Lactococcus *phage sk1 and phage c2 form two closely related lineages, and also the lambda-like Siphoviruses align in a similar way as Rohwer *et al*. However, *Lactococcus *phage r1t is more closely related to *Lactococcus *phage Tuc2009 and TPP901-1 in our alignment than was showed previously. Furthermore, *Acinetobacter *phage AP205 is not as diverged from the other Leviphages in our model as Rohwer shows. Other than that, most phages cluster in the same groups as was shown by Rohwer *et al*, even though we have several more phages and subgroups of phages, resulting in not as distinct groupings as in Rohwer's model. We found that the *P. acnes *phage genomes sequenced so far form a new phylogenetic lineage, well separated from other phages, with no close relatives (see Figure 4 and additional files 3 and 4). Furthermore, the different families of phages are widely distributed over all classes of bacteria, with Siphoviruses being the most abundant family reported in the database. Remarkably, even though PAD20 and PAS50 have a morphology that classifies them as Siphoviruses, some of their closest related phages are *Propionibacterium freudenreichii *phage B5 (inovirus), *Acinetobacter *phage AP205 (Levivirus) and *Pseudomonas *phage phi6 segment L (Cystovirus). Furthermore, a group of podoviruses including *Yersinia *phage Berlin seem to be closely related to a group of myoviruses with *Vibrio*, *Aeromonas *and *Enterobacteria *phages. Also of interest is that most phages infecting *Mycobacterium *and *Streptomyces *were rather closely related to phages from *Propionibacterium*. This is not surprising, since the bacterial hosts also are related. Of more interest is that Proteobacteria such as *Pseudomonas*, *Burkholderia *and *Ralstonia *phages also were related to phages infecting the *Actinobacteria *phylum, while other Proteobacteria as *Vibrio *and *Salmonella *were not. Other than that, bacterial families tend to have closely related phages, even though the classification of the phage families can differ in a bacterial species, as the very closely related *Mycobacterium *phage Rizal and Rosebush, that are classified as a Myovirus and a Siphovirus, respectively. Thus, this clearly shows that classification of phages solely based on morphology is not sufficient to determine to which family a phage should be assigned, but that genomic data is also necessary.

## Conclusions

We have found that phages isolated from *P. acnes *are highly similar and show high nucleotide sequence identity. Several of the phage structural proteins were identified using MALDI-TOF MS/MS. The sequenced phages do not seem to encode any putative virulence factors. Finally we could show that the phages seem to be able to cause pseudolysogeny in their hosts.

## Methods

### Bacteriophages and bacterial isolates

Bacteriophages PAD20 and PAS50 were isolated as previously described [6]. Stock solutions were stored at 4°C in SM-buffer (20 mM Tris-HCl pH 7.5, 100 mM NaCl, 60 mM MgSO_4 _(7H_2_O), 0.01% gelatin). Bacterial isolates KPA171202, AD12, AD20, AD21, AD33 and AS50 were plated from frozen stocks on Tryptic Soy Broth supplemented with 1.5% agar (Bacto, Mt Pritchard, NSW, Australia) and incubated at 37°C under anaerobic conditions until colonies were clearly visible.

### DNA sequencing and analysis

Since we had problems getting high concentrations of pure phage DNA, we adapted a PCR based approach. Phage lysate (10^10 ^PFU/ml) was boiled for 5 min and 1 μl added as a DNA template to Fermentas High Fidelity PCR Enzyme Mix (Vilnius, Lithuania). Primers were designed to generate 4-6 kbp long overlapping products based on the already sequenced *P. acnes *phage PA6 genome, covering the whole genome (see Table 5). The overlapping regions were in most cases more than 200 bp, and confirmatory PCRs were run over the whole genome to exclude that we by this approach missed any inserts. The PCR was run under the following conditions: 94°C 2 min, 10 cycles of 94°C 30 sec, annealing temperature 30 sec, 72°C 6 min. This was followed by 35 repeats of 94°C 30 sec, annealing temperature 30 sec and 72°C 6 min (+20 sec/repeat). PCR products were cleaned up with SpinPrep PCR Clean-up Kit (Novagen, Madison, WI, USA). Products were sequenced using BigDye Terminator v3.1 Cycle Sequencing Kit (Applied Biosystems, Foster City, CA, USA) and analyzed using an ABI 3100 Genetic Analyzer (Applied Biosystems). For each sequencing, a new PCR generated DNA template was used, minimising the impact of the PCR. When assembled, a >3-fold coverage with identical nucleotides was achieved. Sequences were assembled and aligned using the Clustal W algorithm [41]. Analyses were performed using the MacVector v9.5.2 software package (Cary, NC, USA). The sequence of the ssDNA ends was determined by similarity to phage PA6 after a PCR over the ends of the phage genome.

**Table 5:** PCR oligonucleotides used

Name	Sequence (5'-3')
G1 fwd	AGTGAAATACCTCCCTTTTGTGGTTT

G1 rev	TTCTCCTTCACAACAGCAAACGA

G2 fwd	ATTTGTGATGGCTGACGATTTTCTT

G2 rev	AATAAGCATACCAATAACAGGCACCA

G3 fwd	ATTATTGGTATGGTTGCTGGTTTGG

G3 rev	ATCATTGCCGTTCACACCGTC

G4 fwd	TTTGTGTGTGGATGCTCAGCGT

G4 rev	CTACTACAAAACCATCAAACAAGCCAC

G5 fwd	TTCTGTCTGTGTGGTTGAGTGTTTTG

G5 rev	GACAAACTCTCATCCTTCACAAACATTT

G6 fwd	AATTGTAGTCTTTCTTGTGTGGCCC

G6 rev	ATGTTTTTGTGGTTTGTTGTTGGAAT

### Identification of promoter regions and terminator structures

The genome of PAD20, PAS50 and PA6 were screened for putative sigma70-promoters using SAK and BPROM (Softberry, Inc.). Using SAK [42], a value higher than 1 was regarded as positive. Terminator structures were identified using FindTerm (Softberry, Inc.) and EMBOSS Explorer [43]. Palindromes followed by a high content of U and with a stable secondary structure (less than -20.0 in minimum free energy) were considered to be transcriptional terminators.

### Phage protein identification

The phages were purified according to Boulanger [44], with some modifications. Briefly, bacterial lysates were treated with 0.5 M NaCl for 1 h at 4°C to separate phage particles from bacteria. The solution was centrifugated (10 min, 8,000 g, 4°C) and the supernatant moved to a new tube. PEG 6000 was added to a final concentration of 10%, and incubated for 1 h at 4°C. The phages were pelleted by centrifugation at 10,000 g for 15 min at 4°C. The supernatant was discarded and the pellet resuspended in PBS. Whole phage particles were then separated from bacterial proteins using gelfiltration (Superose 6 10/300 GL, GE Healthcare, Sweden, Uppsala) operated on a ÄKTAprime plus (GE Healthcare). Protein fractions containing phage particles were eluted early during the run due to their high molecular mass. Fractions with phage particles were pooled and precipitated with 10% PEG 6000 for 1 h at 4°C. The pellet was resuspended in PBS, mixed with SDS loading buffer, boiled for 3 min and loaded on a 10% SDS-PAGE gel [45]. The gels were run at 170 V for 1 h and stained with 0.25% Coomassie Brilliant Blue. All visible bands were cut out using a sterile scalpel. The gels spots were destained with 50 μ1 50%.acetonitrile (ACN) in 25 mM NH4HCO3 and dried using a speed vacuum for 10-15 min. To the dried spots, 10 mM DTT dissolved in 100 mM NH4HCO3 was added and incubated for 1 hour at 56°C. The DTT was removed and 55 mM iodoacetamide dissolved in NH4HCO3 was added and incubated for 45 min at room temperature in the dark. The gel pieces were washed several times with NH4HCO3, followed by dehydration with ACN and were dried using a speed vacuum. The gel pieces were incubated with 12.5 ng/μl trypsin (Promega) in 50 mM NH4HCO3 at 4°C for 45 minutes. The digestion continued over night at 37°C, and was terminated by the addition of 5% trifluoroacetic acid in 75% ACN. Samples were subjected to HPLC-MS/MS, by analyzing them on a Qtof Ultima API (ESI-MS/MS from Waters, Manchester, UK) coupled to a CapLC (Waters). The peptides were injected and trapped on a pre-column (C18, 300 μm × 5 mm, 5 μm, 100Å, LC-Packings), and separated on a reversed phase analytical column (Atlantis, C18, 75 μm × 150 mm, 3 μm, 100Å, Waters). The flow rate through the column was 250 nl/min. Solvent A was 2% ACN in water with 0.1% formic acid, and solvent B was 90% ACN, 10% water and 0.1% formic acid. The HPLC started with 5% solution B for 5 min, increased the concentration of B to 60% in 40 min, and to 80% B in 5 min. The concentration of B was kept at 80% for 25 min and finally at 5% B for 15 min. The mass spectrometer analysis was made by Data Dependent Acquisition (DDA). The mass range m/z was from 50-1800 for MS/MS. Only spectra from ions with charge states 2 and 3 were acquired. Generated files were used to search in MASCOT MS/MS Ions search using a peptide tolerance of 0.6 Da and a MS/MS tolerance of 0.8 Da. Hits on peptides were regarded positive if they had a p-value below 0.05.

### Serial single-colony isolation

AD20 and AS50 were cultured on Brain Heart Infusion Agar plates (BHIA-plates) for three to four days, until visible single colonies were seen. Ten single colonies from each bacterium were selected, and ten serial streak isolations were made. The presence of phages in the final isolated colonies was detected by a PCR assay screening for the well-conserved major head gene, as described elsewhere [6].

### Growth curve of *P. acnes *isolates

*P. acnes *isolates AD20 and AS50 were plated on BHIA-plates for three to four days. Single colonies were inoculated into pre-reduced BHI broth, and samples were analysed every 12^th ^hour for OD_600_, cfu, infectious phages (pfu_i_), and carriage of phages (pfu_c_). Colony forming units were measured by dilution of the bacterial culture and plating on BHIA-plates. Pfu_i _was measured by sterile filtration of the undiluted growth medium, using overlay plates as described elsewhere [6]. The detection limit using this method was >100 pfu/ml. To investigate the percentage of bacteria carrying phage DNA (pfu_c_), single colonies at each time point were subjected to PCR for the identification of the major head gene, using the recA gene as a positive control, as described elsewhere [6]. All experiments were performed in triplicates.

### Phage phylogenetic analysis

The software Geneious (Geneious Pro 4.6.6, Biomatters Ltd) was used to download 451 different phage genomes from GenBank, searching for genomes with 'phage' in the title. The genomes for *Propionibacterium *phage PAD20 and PAS50 were added, and aligned using the software Mauve [39] with progressive Mauve, using the default settings. The generated guide-tree was then visualized with Geneious. For better visualization, 28 phage genomes were selected based on their localisation and their relationship with *P. acnes *phages on the complete phylogenetic tree, and a new alignment with the same settings was run with only these phages.

### Nucleotide sequence accession number

The GenBank accession numbers for the bacteriophages PAD20 and PAS50 genomes are FJ706171 (protein accession number ACX30796-ACX30840) and FJ706172 (protein accession number ACX30841-ACX30886), respectively.

## Authors' contributions

RL participated in the design of the study, performed the experiments and drafted the manuscript. MC designed the study and revised the manuscript. Both authors read and approved the final manuscript.

## Supplementary Material

Additional file 1 **Nucleotide sequence alignment for gp22 and gp23 in *P*. *acnes *phage PA6, PAD20 and PAS50**. Underlined sequences represent different genes, as indicated by the colour of the line. Phage PAS50 has two internal stop codons (red) in the gene encoding gp22 and will not generate a full-length protein. Phage PAD20 has a deletion in a major part of the gene encoding gp22 resulting in a frame shift and a combined gene gp22.23. Click here for file

Additional file 2 **Gene comparison between phage PA6, PAD20 and PAS50**. An 'x' indicates that the gene is absent, while a minus (-) indicates that no comparison of the genes were possible. Click here for file

Additional file 3 **Bacteriophage genomes used in phylogenetic alignment**. Bacteriophages used in the phylogenetic alignment are ordered together with their accession number for reference. Click here for file

Additional file 4 **Phylogenetic tree of more than 450 bacteriophage genomes**. More than 450 bacteriophage genomes were aligned using progressive Mauve. The generated tree-file was then visualized using the software Geneious The different colours represents different families of phages - bright green (Siphovirus), red (Myovirus), blue (Podovirus), pink (Inovirus), light blue (Levivirus), gray (Cystovirus), purple (Tectivirus), brown (Microvirus), pale green (Corticovirus), orange (Plasmavirus) and black (non-assigned). Click here for file
