# Supplementary figures and images for: Characterization and genome sequencing of two Propionibacterium acnes phages displaying pseudolysogeny
Source: BMC Genomics. 2011 Apr 19;12:198. doi: 10.1186/1471-2164-12-198 (PMC3094311; doi:10.1186/1471-2164-12-198)

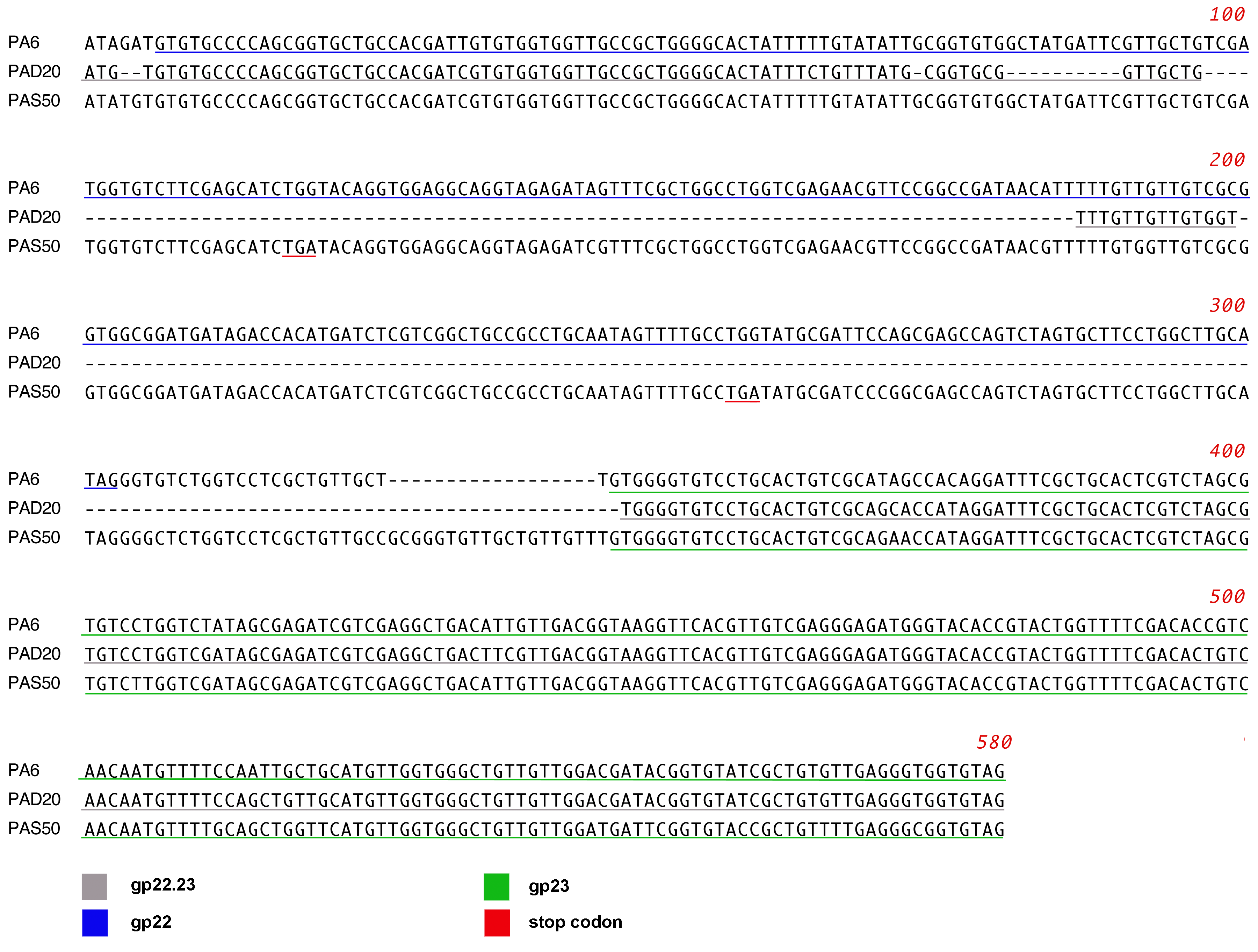

Supplement: Additional file 1 — Nucleotide sequence alignment for gp22 and gp23 in P. acnes phage PA6, PAD20 and PAS50. Underlined sequences represent different genes, as indicated by the colour of the line. Phage PAS50 has two internal stop codons (red) in the gene encoding gp22 and will not generate a full-length protein. Phage PAD20 has a deletion in a major part of the gene encoding gp22 resulting in a frame shift and a combined gene gp22.23. [file 1471-2164-12-198-S1.TIFF]
