# Supplementary material for: Characterization and genome sequencing of two Propionibacterium acnes phages displaying pseudolysogeny
Source: BMC Genomics. 2011 Apr 19;12:198. doi: 10.1186/1471-2164-12-198 (PMC3094311; doi:10.1186/1471-2164-12-198)
Supplement: Additional file 2 — Gene comparison between phage PA6, PAD20 and PAS50. An 'x' indicates that the gene is absent, while a minus (-) indicates that no comparison of the genes were possible. [file 1471-2164-12-198-S2.PDF]

Gene comparison between phage PA6, PAD20 and PAS50

Gene	Length (aa)	PA6/PAD20	PA6/PAS50	PAD20/PAS50
	PA6-PAD20-PAS50	aa id (sim) %	aa id (sim) %	aa id (sim) %
gp1	123-123-123	95.9 (97.6)	96.7 (98.4)	97.6 (97.6)
gp2	503-503-503	97.0 (98.4)	97.0 (98.6)	98.4 (99.8)
gp3	441-441-441	97.1 (98.0)	98.9 (99.3)	97.3 (98.2)
gp4	251-251-251	93.6 (96.4)	95.6 (98.0)	92.4 (96.0)
gp5	184-184-184	95.1 (97.3)	95.1 (97.3)	95.7 (96.7)
gp6	315-315-315	97.5 (98.7)	97.8 (99.0)	97.8 (98.4)
gp7	153-153-153	98.7 (98.7)	99.3 (99.3)	99.3 (99.3)
gp8	115-115-115	93.0 (95.7)	96.5 (98.3)	94.8 (97.4)
gp9	96-96-96	96.9 (99.0)	95.8 (99.0)	99.0 (100)
gp10	115-123-123	94.8 (96.5)	93.9 (96.5)	98.3 (98.3)
gp11	213-209-212	93.0 (94.4)	97.2 (98.1)	94.8 (96.2)
gp12	98-97-98	95.9 (96.9)	96.9 (99.0)	94.9 (95.9)
gp13	95-95-95	96.8 (98.9)	95.8 (97.9)	94.7 (96.8)
gp14	921-921-921	90.9 (95.1)	96.9 (98.3)	91.6 (95.3)
gp15	315-313-313	93.7 (94.9)	94.3 (96.8)	93.9 (96.2)
gp16	385-385-385	96.4 (98.2)	96.1 (97.4)	95.1 (96.6)
gp17	272-272-272	89.7 (94.5)	93.4 (97.4)	93.0 (96.0)
gp18	79-79-81	94.9 (98.7)	93.8 (96.3)	91.4 (95.1)
gp19	246-246-220	67.1 (71.0)	77.4 (83.4)	67.9 (73.9)
gp20	286-287-285	91.3 (95.1)	95.1 (97.9)	89.9 (95.1)
gp21	133-134-133	84.3 (90.3)	94.0 (97.7)	82.8 (89.6)
gp22	98-x-x	-	-	-
gp22.23	x-109-x	-	-	-
gp23	77-x-77	92.2 (94.8)	87.0 (89.6)	88.3 (92.2)
gp24	130-131-129	85.6 (90.2)	90.0 (93.1)	89.4 (93.9)
gp25	91-93-94	90.3 (91.4)	90.4 (91.5)	85.4 (89.6)
gp26	106-106-106	91.5 (92.5)	97.2 (98.1)	93.4 (94.3)
gp27	348-348-348	92.2 (96.3)	89.4 (95.1)	95.4 (98.3)
gp28	64-64-64	82.8 (95.3)	82.8 (93.8)	98.4 (100)
gp29.1	188-187-187	84.0 (90.4)	84.0 (90.4)	99.5 (99.5)
gp30	187-187-187	87.2 (94.7)	88.2 (94.7)	94.7 (97.9)
gp31	223-223-222	94.6 (96.0)	93.3 (94.6)	96.0 (96.9)
gp32	133-133-133	94.0 (96.2)	93.2 (96.2)	95.5 (97.0)
gp33	118-118-118	91.5 (94.9)	92.4 (95.8)	99.2 (99.2)
gp34	317-287-287	96.2 (96.9)	94.1 (94.8)	97.6 (97.6)
gp35	156-154-154	90.4 (92.9)	91.7 (93.6)	98.7 (99.4)
gp36	136-136-136	80.1 (89.7)	77.2 (86.8)	96.3 (96.3)
gp37	315-309-343	89.0 (95.5)	90.0 (95.1)	91.9 (94.5)
gp38	119-117-117	84.9 (89.1)	89.9 (95.0)	83.8 (92.3)
gp39	67-67-67	95.5 (98.5)	95.5 (98.5)	100 (100)

gp40	75-75-75	73.3 (84.0)	73.3 (84.0)	100 (100)
gp41	179-177-177	76.5 (87.2)	81.0 (87.7)	92.7 (96.6)
gp42	95-x-94	-	75.8 (86.3)	-
gp43	103-103-103	83.5 (89.3)	94.2 (97.1)	81.6 (88.3)
gp44	88-88-88	81.8 (83.0)	81.8 (83.0)	100 (100)
gp45	60-x-x	-	-	-
gp46	51-51-51	84.3 (88.2)	88.2 (90.2)	94.1 (96.1)
gp47	59-44-44	32.9 (35.7)	32.9 (35.7)	100 (100)
gp48	100-100-101	96.0 (98.0)	93.1 (98.0)	95.0 (98.0)
gp49	117-160-160	93.1 (95.7)	93.1 (96.6)	94.8 (95.7)
gp50	130-130-130	90.8 (93.1)	93.1 (95.4)	91.5 (91.5)
