# Supplementary material for: Characterization and genome sequencing of two Propionibacterium acnes phages displaying pseudolysogeny
Source: BMC Genomics. 2011 Apr 19;12:198. doi: 10.1186/1471-2164-12-198 (PMC3094311; doi:10.1186/1471-2164-12-198)
Supplement: Additional file 3 — Bacteriophage genomes used in phylogenetic alignment. Bacteriophages used in the phylogenetic alignment are ordered together with their accession number for reference. [file 1471-2164-12-198-S3.PDF]

Bacteriophage	Accession number
Acholeplasma phage L2	NC_001447.1
Acholeplasma phage MV-L1	NC_001341
Acinetobacter phage AP205	NC_002700
Actinomyces phage Av-1	NC_009643.1
Actinoplanes phage phiAsp2	NC_005885.1
Aeromonas phage 25	NC_008208.1
Aeromonas phage 31	NC_007022.1
Aeromonas phage 44RR2.8t	NC_005135.1
Aeromonas phage Aeh1	NC_005260.1
Aeromonas phage phi018P	NC_009542.2
Azospirillum phage Cd	NC_010355.1
Bacillus phage 0305phi8-36	NC_009760.1
Bacillus phage AP50	NC_011523.1
Bacillus phage B103	NC_004165.1
Bacillus phage Bam35c	NC_005258.1
Bacillus phage BCJA1c	NC_006557.1
Bacillus phage Cherry	NC_007457.1
Bacillus phage Fah	NC_007814.1
Bacillus phage GA-1	NC_002649.1
Bacillus phage Gamma	NC_007458.1
Bacillus phage GIL16c	NC_006945.1
Bacillus phage IEBH	NC_011167.1
Bacillus phage phi29	NC_011048.1
Bacillus phage phi105	NC_004167.1
Bacillus phage SPBc2	NC_001884.1
Bacillus phage SPO1	NC_011421.1
Bacillus phage SPP1	NC_004166.2
Bacillus phage TP21-L	NC_011645.1
Bacillus phage WBeta	NC_007734.1
Bacteroides phage B40-8	NC_011222.1
Bdellovibrio phage phiMH2K	NC_002643
Bordetella phage BIP-1	NC_005809.1
Bordetella phage BMP-1	NC_005808.1
Bordetella phage BPP-1	NC_005357.1
Burkholderia ambifaria phage BcepF1	NC_009015.1
Burkholderia phage Bcep1	NC_005263.2
Burkholderia phage Bcep22	NC_005262.2
Burkholderia phage Bcep43	NC_005342.2
Burkholderia phage Bcep176	NC_007497.1
Burkholderia phage Bcep781	NC_004333.2
Burkholderia phage BcepB1A	NC_005886.2
Burkholderia phage BcepC6B	NC_005887.1
Burkholderia phage BcepGomr	NC_009447.1
Burkholderia phage BcepMu	NC_005882.1
Burkholderia phage BcepNazgul	NC_005091.2
Burkholderia phage BcepNY3	NC_009604.1
Burkholderia phage KS10	NC_011216.1
Burkholderia phage phi644-2	NC_009235.2
Burkholderia phage phi1026b	NC_005284.1
Burkholderia phage phiE12-2	NC_009236.1
Burkholderia phage phiE125	NC_003309.1
Burkholderia phage phiE202	NC_009234.1
Burkholderia phage phiE255	NC_009237.1
Chlamydia phage 3	NC_008355
Chlamydia phage 4	NC_007461
Chlamydia phage Chp1	NC_001741
Chlamydia phage Chp2	NC_002194

Chlamydia phage phiCPAR39	NC_002180
Chlamydia phage phiCPG1	NC_001998
Clostridium phage 39-O	NC_011318.1
Clostridium phage c-st	NC_007581.1
Clostridium phage phi3626	NC_003524.1
Clostridium phage phiC2	NC_009231.1
Clostridium phage phiCD27	NC_011398.1
Clostridium phage phiCD119	NC_007917.1
Clostridium phage phiSM101	NC_008265.1
Corynebacterium phage BFK20	NC_009799.2
Corynebacterium phage P1201	NC_009816.1
Enterobacteria phage 13a	NC_011045.1
Enterobacteria phage 933W	NC_000924.1
Enterobacteria phage alpha3	NC_001330
Enterobacteria phage BA14	NC_011040.1
Enterobacteria phage BP-4795	NC_004813.1
Enterobacteria phage EcoDS1	NC_011042.1
Enterobacteria phage EPS7	NC_010583.1
Enterobacteria phage epsilon15	NC_004775.1
Enterobacteria phage ES18	NC_006949.1
Enterobacteria phage Felix01	NC_005282.1
Enterobacteria phage Fels-2	NC_010463.1
Enterobacteria phage FI	NC_004301
Enterobacteria phage G4	NC_001420
Enterobacteria phage GA	NC_001426
Enterobacteria phage HK022	NC_002166.1
Enterobacteria phage HK97	NC_002167.1
Enterobacteria phage HK620	NC_002730.1
Enterobacteria phage I2-2	NC_001332
Enterobacteria phage ID2 Moscow/ID/2001	NC_007817
Enterobacteria phage ID18	NC_007856
Enterobacteria phage If1	NC_001954
Enterobacteria phage Ike	NC_002014
Enterobacteria phage JK06	NC_007291.1
Enterobacteria phage JS98	NC_010105.1
Enterobacteria phage K1-5	NC_008152.1
Enterobacteria phage K1E	NC_007637.1
Enterobacteria phage K1F	NC_007456.1
Enterobacteria phage lambda	NC_001416.1
Enterobacteria phage M13	NC_003287
Enterobacteria phage Min27	NC_010237.1
Enterobacteria phage MS2	NC_001417
Enterobacteria phage Mu	NC_000929.1
Enterobacteria phage N4	NC_008720.1
Enterobacteria phage N15	NC_001901.1
Enterobacteria phage P1	NC_005856.1
Enterobacteria phage P2	NC_001895.1
Enterobacteria phage P4	NC_001609.1
Enterobacteria phage P22	NC_002371.2
Enterobacteria phage Phi1	NC_009821.1
Enterobacteria phage phiEco32	NC_010324.1
Enterobacteria phage phiEcoM-GJ1	NC_010106.1
Enterobacteria phage phiP27	NC_003356.1
Enterobacteria phage phiV10	NC_007804.2
Enterobacteria phage phiX174	NC_001422
Enterobacteria phage PRD1	NC_001421.2
Enterobacteria phage PsP3	NC_005340.1
Enterobacteria phage Qbeta	NC_001890

Enterobacteria phage RB32	NC_008515.1
Enterobacteria phage RB43	NC_007023.1
Enterobacteria phage RB49	NC_005066.1
Enterobacteria phage RB69	NC_004928.1
Enterobacteria phage RTP	NC_007603.1
Enterobacteria phage Sf6	NC_005344.1
Enterobacteria phage SfV	NC_003444.1
Enterobacteria phage SP6	NC_004831.2
Enterobacteria phage ST64T	NC_004348.1
Enterobacteria phage ST104	NC_005841.1
Enterobacteria phage T1	NC_005833.1
Enterobacteria phage T3	NC_003298.1
Enterobacteria phage T4	NC_000866.4
Enterobacteria phage T5	NC_005859.1
Enterobacteria phage T7	NC_001604.1
Enterobacteria phage TLS	NC_009540.1
Enterobacteria phage VT2-Sakai	NC_000902.1
Enterobacteria phage WA13	NC_007821
Enterobacteria phage YYZ-2008	NC_011356.1
Enterococcus phage phiEF24C	NC_009904.1
Erwinia phage Era103	NC_009014.1
Erwinia phage phiEa21-4	NC_011811.1
Escherichia phage rv5	NC_011041.1
Flavobacterium phage 11b	NC_006356.2
Geobacillus phage GBSV1	NC_008376.2
Haemophilus phage HP1	NC_001697.1
Haemophilus phage HP2	NC_003315.1
Haloarcula phage SH1	NC_007217.1
Halomonas phage phiHAP-1	NC_010342.1
Klebsiella phage K11	NC_011043.1
Klebsiella phage phiKO2	NC_005857.1
Kluyvera phage Kvp1	NC_011534.1
Lactobacillus phage A2	NC_004112.1
Lactobacillus phage KC5a	NC_007924.1
Lactobacillus phage Lc-Nu	NC_007501.1
Lactobacillus phage LL-H	NC_009554.1
Lactobacillus phage LP65	NC_006565.1
Lactobacillus phage Lrm1	NC_011104.1
Lactobacillus phage Lv-1	NC_011801.1
Lactobacillus phage phiAT3	NC_005893.1
Lactobacillus phage phig1e	NC_004305.1
Lactobacillus phage phiJl-1	NC_006936.1
Lactococcus phage 712	NC_008370.1
Lactococcus phage 1706	NC_010576.1
Lactococcus phage asccphi28	NC_010363.1
Lactococcus phage bIBB29	NC_011046.1
Lactococcus phage bIL67	NC_001629.1
Lactococcus phage bIL170	NC_001909.1
Lactococcus phage BK5-T	NC_002796.1
Lactococcus phage c2	NC_001706.1
Lactococcus phage jj50	NC_008371.1
Lactococcus phage KSY1	NC_009817.1
Lactococcus phage P008	NC_008363.1
Lactococcus phage P4268	NC_004746.1
Lactococcus phage phiLC3	NC_005822.1
Lactococcus phage Q54	NC_008364.1
Lactococcus phage r1t	NC_004302.1
Lactococcus phage sk1	NC_001835.1

Lactococcus phage TP901-1	NC_002747.1
Lactococcus phage Tuc2009	NC_002703.1
Lactococcus phage ul36	NC_004066.1
Leuconostoc phage L5	NC_009534
Listeria phage A006	NC_009815.1
Listeria phage A118	NC_003216.1
Listeria phage A500	NC_009810.1
Listeria phage A511	NC_009811.2
Listeria phage B025	NC_009812.1
Listeria phage B054	NC_009813.1
Listeria phage P35	NC_009814.1
Listeria phage P40	NC_011308.1
Listonella phage phiHSIC	NC_006953.1
Mannheimia phage phiMHaA1	NC_008201.1
Methanobacterium phage psiM2	NC_001902.1
Microbacterium phage Min1	NC_009603.1
Microcystis phage Ma-LMM01	NC_008562.1
Morganella phage MmP1	NC_011085.1
Mycobacterium phage 244	NC_008194.1
Mycobacterium phage Adjutor	NC_010763.1
Mycobacterium phage Barnyard	NC_004689.1
Mycobacterium phage Bethlehem	NC_009878.1
Mycobacterium phage Boomer	NC_011054.1
Mycobacterium phage BPs	NC_010762.1
Mycobacterium phage Brujita	NC_011291.1
Mycobacterium phage Butterscotch	NC_011286.1
Mycobacterium phage Bxb1	NC_002656.1
Mycobacterium phage BXz1	NC_004687.1
Mycobacterium phage BXz2	NC_004682.1
Mycobacterium phage Cali	NC_011271.1
Mycobacterium phage Catera	NC_008207.1
Mycobacterium phage Chah	NC_011284.1
Mycobacterium phage Che8	NC_004680.1
Mycobacterium phage Che9c	NC_004683.1
Mycobacterium phage Che9d	NC_004686.1
Mycobacterium phage Che12	NC_008203.1
Mycobacterium phage Cjw1	NC_004681.1
Mycobacterium phage Cooper	NC_008195.1
Mycobacterium phage Corndog	NC_004685.1
Mycobacterium phage D29	NC_001900.1
Mycobacterium phage DD5	NC_011022.1
Mycobacterium phage Fruitloop	NC_011288.1
Mycobacterium phage Giles	NC_009993.1
Mycobacterium phage Gumball	NC_011290.1
Mycobacterium phage Halo	NC_008202.1
Mycobacterium phage Jasper	NC_011020.1
Mycobacterium phage KBG	NC_011019.1
Mycobacterium phage Konstantine	NC_011292.1
Mycobacterium phage Kostya	NC_011056.1
Mycobacterium phage L5	NC_001335.1
Mycobacterium phage Llij	NC_008196.1
Mycobacterium phage Lockley	NC_011021.1
Mycobacterium phage Myrna	NC_011273.1
Mycobacterium phage Nigel	NC_011044.1
Mycobacterium phage Omega	NC_004688.1
Mycobacterium phage Orion	NC_008197.1
Mycobacterium phage Pacc40	NC_011287.1
Mycobacterium phage PBI1	NC_008198.1

Mycobacterium phage PG1	NC_005259.1
Mycobacterium phage Phaedrus	NC_011057.1
Mycobacterium phage Phlyer	NC_012027.1
Mycobacterium phage Pipefish	NC_008199.1
Mycobacterium phage PLot	NC_008200.1
Mycobacterium phage PMC	NC_008205.1
Mycobacterium phage Porky	NC_011055.1
Mycobacterium phage Predator	NC_011039.1
Mycobacterium phage Pukovnik	NC_011023.1
Mycobacterium phage Qyrzula	NC_008204.1
Mycobacterium phage Ramsey	NC_011289.1
Mycobacterium phage Rizal	NC_011272.1
Mycobacterium phage Rosebush	NC_004684.1
Mycobacterium phage ScottMcG	NC_011269.1
Mycobacterium phage Solon	NC_011267.1
Mycobacterium phage Spud	NC_011270.1
Mycobacterium phage TM4	NC_003387.1
Mycobacterium phage Troll4	NC_011285.1
Mycobacterium phage Tweety	NC_009820.1
Mycobacterium phage U2	NC_009877.1
Mycobacterium phage Wildcat	NC_008206.1
Mycoplasma phage MAV1	NC_001942.1
Mycoplasma phage P1	NC_002515.1
Mycoplasma phage phiMFV1	NC_005964.1
Myxococcus phage Mx8	NC_003085.1
Natrialba phage PhiCh1	NC_004084.1
Pasteurella phage F108	NC_008193.1
Phage cdtI	NC_009514.1
Phage Gifsy-1	NC_010392.1
Phage Gifsy-2	NC_010393.1
Phage phiJL001	NC_006938.1
Phormidium phage Pf-WMP3	NC_009551.1
Phormidium phage Pf-WMP4	NC_008367.1
Prochlorococcus phage P-SSM2	NC_006883.1
Prochlorococcus phage P-SSM4	NC_006884.1
Prochlorococcus phage P-SSP7	NC_006882.1
Propionibacterium phage B5	NC_003460
Propionibacterium phage PA6	NC_009541.1
Propionibacterium phage PAD20	FJ706171
Propionibacterium phage PAS50	FJ706172
Pseudoalteromonas phage PM2	NC_000867.1
Pseudomonas phage 14-1	NC_011703.1
Pseudomonas phage 73	NC_007806.1
Pseudomonas phage 119X	NC_007807.1
Pseudomonas phage 201phi2-1	NC_010821.1
Pseudomonas phage B3	NC_006548.1
Pseudomonas phage D3	NC_002484.1
Pseudomonas phage D3112	NC_005178.1
Pseudomonas phage DMS3	NC_008717.1
Pseudomonas phage EL	NC_007623.1
Pseudomonas phage F8	NC_007810.1
Pseudomonas phage F10	NC_007805.1
Pseudomonas phage F116	NC_006552.1
Pseudomonas phage gh-1	NC_004665.1
Pseudomonas phage LBL3	NC_011165.1
Pseudomonas phage LKA1	NC_009936.1
Pseudomonas phage LKD16	NC_009935.1
Pseudomonas phage LMA2	NC_011166.1

Pseudomonas phage LUZ19	NC_010326.1
Pseudomonas phage LUZ24	NC_010325.1
Pseudomonas phage M6	NC_007809.1
Pseudomonas phage MP22	NC_009818.1
Pseudomonas phage MP29	NC_011613.1
Pseudomonas phage MP38	NC_011611.1
Pseudomonas phage PA11	NC_007808.1
Pseudomonas phage PAJU2	NC_011373.1
Pseudomonas phage PaP2	NC_005884.1
Pseudomonas phage PaP3	NC_004466.2
Pseudomonas phage PB1	NC_011810.1
Pseudomonas phage Pf1	NC_001331
Pseudomonas phage Pf3	NC_001418
Pseudomonas phage phi6 segment L	NC_003715
Pseudomonas phage phi6 segment M	NC_003716
Pseudomonas phage phi6 segment S	NC_003714
Pseudomonas phage phi8 segment L	NC_003299
Pseudomonas phage phi8 segment M	NC_003300
Pseudomonas phage phi8 segment S	NC_003301
Pseudomonas phage phi12 segment L	NC_004173
Pseudomonas phage phi12 segment M	NC_004175
Pseudomonas phage phi12 segment S	NC_004174
Pseudomonas phage phi13 segment L	NC_004172
Pseudomonas phage phi13 segment M	NC_004171
Pseudomonas phage phi13 segment S	NC_004170
Pseudomonas phage phi2954 segment L	NC_012091
Pseudomonas phage phi2954 segment M	NC_012092
Pseudomonas phage phi2954 segment S	NC_012093
Pseudomonas phage phiCTX	NC_003278.1
Pseudomonas phage phiKMV	NC_005045.1
Pseudomonas phage phiKZ	NC_004629.1
Pseudomonas phage PP7	NC_001628
Pseudomonas phage PRR1	NC_008294
Pseudomonas phage PT2	NC_011107.1
Pseudomonas phage PT5	NC_011105.1
Pseudomonas phage SN	NC_011756.1
Pseudomonas phage YuA	NC_010116.1
Ralstonia phage p12J	NC_005131
Ralstonia phage phiRSA1	NC_009382.1
Ralstonia phage RSB1	NC_011201.1
Ralstonia phage RSL1	NC_010811.1
Ralstonia phage RSM1	NC_008574
Ralstonia phage RSM3	NC_011399
Ralstonia phage RSS1	NC_008575
Rhizobium phage 16-3	NC_011103.1
Rhodothermus phage RM378	NC_004735.1
Roseobacter phage SIO1	NC_002519.1
Salmonella phage E1	NC_010495.1
Salmonella phage epsilon34	NC_011976.1
Salmonella phage Fels-1	NC_010391.1
Salmonella phage KS7	NC_006940.1
Salmonella phage phiSG-JL2	NC_010807.1
Salmonella phage SETP3	NC_009232.1
Salmonella phage ST64B	NC_004313.1
Sinorhizobium phage PBC5	NC_003324.1
Sodalis phage phiSG1	NC_007902.1
Spiroplasma phage 1-C74	NC_003793
Spiroplasma phage 1-R8A2B	NC_001365

Spiroplasma phage 4	NC_003438
Spiroplasma phage SVTS2	NC_001270
Staphylococcus aureus phage P68	NC_004679.1
Staphylococcus phage 3A	NC_007053.1
Staphylococcus phage 29	NC_007061.1
Staphylococcus phage 37	NC_007055.1
Staphylococcus phage 42E	NC_007052.1
Staphylococcus phage 44AHJD	NC_004678.1
Staphylococcus phage 47	NC_007054.1
Staphylococcus phage 52A	NC_007062.1
Staphylococcus phage 53	NC_007049.1
Staphylococcus phage 55	NC_007060.1
Staphylococcus phage 66	NC_007046.1
Staphylococcus phage 69	NC_007048.1
Staphylococcus phage 71	NC_007059.1
Staphylococcus phage 77	NC_005356.1
Staphylococcus phage 80alpha	NC_009526.1
Staphylococcus phage 85	NC_007050.1
Staphylococcus phage 88	NC_007063.1
Staphylococcus phage 92	NC_007064.1
Staphylococcus phage 96	NC_007057.1
Staphylococcus phage 187	NC_007047.1
Staphylococcus phage 2638A	NC_007051.1
Staphylococcus phage CNPH82	NC_008722.1
Staphylococcus phage EW	NC_007056.1
Staphylococcus phage G1	NC_007066.1
Staphylococcus phage K	NC_005880.1
Staphylococcus phage PH15	NC_008723.1
Staphylococcus phage phi2958PVL	NC_011344.1
Staphylococcus phage phiETA	NC_003288.1
Staphylococcus phage phiETA2	NC_008798.1
Staphylococcus phage phiETA3	NC_008799.1
Staphylococcus phage phiMR11	NC_010147.1
Staphylococcus phage phiMR25	NC_010808.1
Staphylococcus phage phiNM1	NC_008583.1
Staphylococcus phage phiPVL108	NC_008689.1
Staphylococcus phage phiSauS-IPLA35	NC_011612.1
Staphylococcus phage phiSauS-IPLA88	NC_011614.1
Staphylococcus phage phiSLT	NC_002661.2
Staphylococcus phage PT1028	NC_007045.1
Staphylococcus phage ROSA	NC_007058.1
Staphylococcus phage SAP-2	NC_009875.1
Staphylococcus phage Twort	NC_007021.1
Staphylococcus phage X2	NC_007065.1
Stenotrophomonas phage phiSMA9	NC_007189
Stenotrophomonas phage S1	NC_011589.1
Streptococcus phage 858	NC_010353.1
Streptococcus phage 2972	NC_007019.1
Streptococcus phage 7201	NC_002185.1
Streptococcus phage C1	NC_004814.1
Streptococcus phage Cp-1	NC_001825.1
Streptococcus phage DT1	NC_002072.2
Streptococcus phage O1205	NC_004303.1
Streptococcus phage P9	NC_009819.1
Streptococcus phage PH15	NC_010945.1
Streptococcus phage phi3396	NC_009018.1
Streptococcus phage Sfi11	NC_002214.1
Streptococcus phage Sfi19	NC_000871.1

Streptococcus phage Sfi21	NC_000872.1
Streptococcus phage SMP	NC_008721.1
Streptomyces phage mu1/6	NC_007967.1
Streptomyces phage phiBT1	NC_004664.2
Streptomyces phage phiC31	NC_001978.2
Streptomyces phage VWB	NC_005345.2
Stx1 converting phage	NC_004913.1
Stx2 converting phage 86	NC_008464.1
Stx2 converting phage 1717	NC_011357.1
Stx2 converting phage I	NC_003525.1
Stx2 converting phage II	NC_004914.1
Synechococcus phage P60	NC_003390.1
Synechococcus phage S-PM2	NC_006820.1
Synechococcus phage syn9	NC_008296.2
Temperate phage phiNIH1.1	NC_003157.4
Thalassomonas phage BA3	NC_009990.1
Thermus phage IN93	NC_004462.1
Thermus phage P23-45	NC_009803.1
Thermus phage P74-26	NC_009804.1
Thermus phage phiYS40	NC_008584.1
Vibrio phage fs1	NC_004306
Vibrio phage fs2	NC_001956
Vibrio phage K139	NC_003313.1
Vibrio phage kappa	NC_010275.1
VIbrio phage KSF-1phi	NC_006294
Vibrio phage KVP40	NC_005083.2
Vibrio phage Vf03K6	NC_002362
Vibrio phage Vf04K68	NC_002363
Vibrio phage Vf12	NC_005949
Vibrio phage Vf33	NC_005948
Vibrio phage VGJphi	NC_004736
Vibrio phage VHML	NC_004456.1
Vibrio phage VP2	NC_005879.1
Vibrio phage VP5	NC_005891.1
Vibrio phage VP882	NC_009016.1
Vibrio phage VSK	NC_003327
Xanthomonas phage Cf1c	NC_001396
Xanthomonas phage OP1	NC_007709.1
Xanthomonas phage OP2	NC_007710.1
Xanthomonas phage Xop411	NC_009543.1
Xanthomonas phage Xp10	NC_004902.1
Xanthomonas phage Xp15	NC_007024.1
Yersinia pestis phage phiA1122	NC_004777.1
Yersinia phage Berlin	NC_008694.1
Yersinia phage L-413C	NC_004745.1
Yersinia phage phiYe03	NC_001271.1
Yersinia phage PY54	NC_005069.1
Yersinia phage Yepe2	NC_011038.1
